# Efficient suppression of parkinsonian beta oscillations in a closed-loop model of deep brain stimulation with amplitude modulation

**DOI:** 10.3389/fnhum.2022.1013155

**Published:** 2023-01-26

**Authors:** Fatemeh Bahadori-Jahromi, Sina Salehi, Mojtaba Madadi Asl, Alireza Valizadeh

**Affiliations:** ^1^Department of Physics, Institute for Advanced Studies in Basic Sciences (IASBS), Zanjan, Iran; ^2^Shiraz Neuroscience Research Center, Shiraz University of Medical Sciences, Shiraz, Iran; ^3^School of Biological Sciences, Institute for Research in Fundamental Sciences (IPM), Tehran, Iran; ^4^Pasargad Institute for Advanced Innovative Solutions (PIAIS), Tehran, Iran

**Keywords:** beta oscillation, Parkinson's disease, closed-loop deep brain stimulation, amplitude modulation, synchronization

## Abstract

**Introduction:**

Parkinson's disease (PD) is a movement disorder characterized by the pathological beta band (15–30 Hz) neural oscillations within the basal ganglia (BG). It is shown that the suppression of abnormal beta oscillations is correlated with the improvement of PD motor symptoms, which is a goal of standard therapies including deep brain stimulation (DBS). To overcome the stimulation-induced side effects and inefficiencies of conventional DBS (cDBS) and to reduce the administered stimulation current, closed-loop adaptive DBS (aDBS) techniques were developed. In this method, the frequency and/or amplitude of stimulation are modulated based on various disease biomarkers.

**Methods:**

Here, by computational modeling of a cortico-BG-thalamic network in normal and PD conditions, we show that closed-loop aDBS of the subthalamic nucleus (STN) with amplitude modulation leads to a more effective suppression of pathological beta oscillations within the parkinsonian BG.

**Results:**

Our results show that beta band neural oscillations are restored to their normal range and the reliability of the response of the thalamic neurons to motor cortex commands is retained due to aDBS with amplitude modulation. Furthermore, notably less stimulation current is administered during aDBS compared with cDBS due to a closed-loop control of stimulation amplitude based on the STN local field potential (LFP) beta activity.

**Discussion:**

Efficient models of closed-loop stimulation may contribute to the clinical development of optimized aDBS techniques designed to reduce potential stimulation-induced side effects of cDBS in PD patients while leading to a better therapeutic outcome.

## 1. Introduction

Parkinson's disease (PD) is a neurodegenerative movement disorder characterized by abnormal neural oscillations in the beta band (15–30 Hz) frequency within the basal ganglia (BG) (Brown et al., 2001; Hammond et al., 2007; Mallet et al., 2008; Asadi et al., 2022). The BG circuitry is massively modulated by dopamine (DA) released from dopaminergic (DAergic) neurons in the substantia nigra pars compacta (SNc). Significant degeneration of DAergic neurons triggers a cascade of maladaptive or compensatory changes within the BG (Blandini et al., 2000; Madadi Asl et al., 2022b), ultimately resulting in the emergence of pathological patterns of activity and connectivity observed in experimental PD models (Galvan et al., 2015; Madadi Asl et al., 2022b). Particularly, striatal inhibition in the direct pathway is suppressed following DA loss, whereas it is enhanced in the indirect pathway (Lemos et al., 2016). As a result, the inhibitory control of globus pallidus externus (GPe) over subthalamic nucleus (STN) reduced (Fan et al., 2012; Madadi Asl et al., 2022a) and excessive beta oscillations emerged (Brown et al., 2001; Hammond et al., 2007; Mallet et al., 2008; Asadi et al., 2022). Finally, globus pallidus internus (GPi) receives more excitatory drive leading to an enhanced inhibition of the thalamo-cortical circuits, which contributes to motor dysfunction in PD (DeLong, 1990; Graybiel et al., 1994).

It is shown that the reduction of pathological beta oscillations is correlated with improved motor performance in PD (Meissner et al., 2005; Kühn et al., 2006, 2008). High-frequency (>100 Hz) deep brain stimulation (HF-DBS) is the standard clinical therapy for medically refractory PD (Benabid, 2003; Benabid et al., 2009). In a conventional DBS (cDBS) protocol, a train of electrical pulses is continuously administered to the target structure, for example, the STN using chronically implanted depth electrodes (Benabid, 2003; Benabid et al., 2009). HF-DBS may cause side effects, such as dysarthria, dysesthesia, and cerebellar ataxia (Volkmann, 2004; Baizabal-Carvallo and Jankovic, 2016). On the other hand, some patients with PD may show unsatisfactory outcomes despite proper electrode placement (Limousin et al., 1999). This led to the pre-clinical and clinical testing of closed-loop and on-demand adaptive DBS (aDBS) (Little et al., 2013, 2016; Priori et al., 2013; Rosa et al., 2015, 2017; Johnson et al., 2016; Piña-Fuentes et al., 2017; Tinkhauser et al., 2017; Guidetti et al., 2021) for a more effective control of pathological beta band oscillatory activity.

In a closed-loop aDBS configuration, the patient's clinical state is assessed and utilized to adjust stimulation parameters, that is, to modify the frequency and/or amplitude of stimulation in a state-dependent manner (Daneshzand et al., 2018; Popovych and Tass, 2019; Fleming et al., 2020b). This can ultimately reduce possible side effects by reducing the amount of administered stimulation current (Pyragas et al., 2020). The modulation of stimulation parameters in closed-loop approaches is realized based on specific biomarkers that are used to estimate the symptom severity. One of the appealing biomarkers for closed-loop DBS in PD is the power of beta band oscillatory activity in the STN local field potential (LFP) that has been utilized in several variations of aDBS protocols addressed both in computational (Tukhlina et al., 2007; Popovych et al., 2017b; Popovych and Tass, 2019; Fleming et al., 2020a,b) and experimental (Little et al., 2013; Rosa et al., 2015; Arlotti et al., 2018; Velisar et al., 2019) studies.

One of the first closed-loop strategies tested in patients with PD was the on–off stimulation strategy where stimulation is turned on and off depending on whether the biomarker exceeded a predefined threshold (Little et al., 2013, 2016). More specifically, aDBS of the STN in patients with advanced PD improved motor symptoms by 66%, which were 29% better than cDBS, despite delivering ≲ 50% less current than cDBS. These improvements were achieved with a 56% reduction in stimulation time compared with cDBS (Little et al., 2013). In comparison with the open-loop stimulation, the on–off stimulation strategy can be more effective in suppressing abnormal oscillations in patients with PD; however, its effectiveness is limited by the fixed choice of stimulation parameters (Little et al., 2013, 2016), as in open-loop cDBS. Later, a dual threshold strategy was introduced that modifies the amplitude of stimulation to confine the biomarker within the desired range (Velisar et al., 2019). Alternatively, stimulation strategies employing proportional amplitude modulation, in which the DBS amplitude is proportional to the measured biomarker (e.g., LFP beta band activity), can be, in principle, more beneficiary as demonstrated both computationally (Tukhlina et al., 2007; Popovych and Tass, 2019) and clinically (Rosa et al., 2015; Arlotti et al., 2018). Indeed, the adjustment of stimulation amplitude based on slowly varying beta activity is not only well-tolerated by patients but also can effectively reduce pathological beta oscillations to improve PD symptoms (Rosa et al., 2015; Arlotti et al., 2018).

In the context of amplitude modulation stimulation strategies, control theory incorporates a variety of schemes that may be more efficient in suppressing PD symptoms, while reducing the amount of delivered current. Development and testing of effective control schemes for DBS in a clinical situation are challenging due to the invasive nature of DBS surgery. Alternatively, computational modeling offers a suitable framework for designing and testing different versions of closed-loop DBS control (Goldobin et al., 2003; Rosenblum and Pikovsky, 2004; Gorzelic et al., 2013; Popovych et al., 2017a; Popovych and Tass, 2019; Su et al., 2019; Fleming et al., 2020a,b). For example, adaptive pulsatile linear delayed feedback stimulation (apLDF) with on–off delivery can induce desynchronization in pathologically synchronized network models (Popovych and Tass, 2019). Interestingly, introducing interphase gap between the stimulation pulses can significantly improve the stimulation-induced desynchronization (Popovych et al., 2017b). Recent computational studies employed clinically viable control schemes for amplitude and frequency modulation, for example, proportional (P) and proportional–integral (PI) closed-loop controllers to suppress PD-related pathological beta activity with a reduced amount of delivered stimulation current in simple network models (Fleming et al., 2020a,b; Weerasinghe et al., 2021). Other closed-loop computational approaches such as phase-specific aDBS, whereby the stimulation is locked to a particular phase of tremor, have been shown to improve therapeutic efficacy (Toth and Wilson, 2022). Specifically, near-periodic phase-specific aDBS can effectively disrupt excessive synchronization in large populations of oscillatory neurons caused by strong coupling.

In this study, our aim was to present a simple, yet comprehensive bio-inspired model of the cortico-BG-thalamic network comprising cortex, striatal D1 and D2 medium spiny neurons (MSNs), GPe, globus pallidus internus (GPi), STN, and thalamus. A more complete set of the BG nuclei used here improves the model predictions and its accuracy. Specifically, we set the model parameters in a way that the dynamics of the network were similar to those reported experimentally for normal and PD states (Holgado et al., 2010; Pavlides et al., 2015). Then, we administered high-frequency stimulation to the parkinsonian STN in our model and investigated its effect on the pathological beta oscillations within the BG. First, we used a cDBS protocol where stimulation pulses were continuously delivered to the STN with a fixed frequency and amplitude. To improve the beta suppression efficiency while consuming less stimulation current, we then used an aDBS protocol that employed the same stimulation frequency but with a closed-loop feedback control of stimulation amplitude based on the STN beta activity.

Our results show that aDBS protocol can effectively suppress abnormal beta oscillations within the BG and preserve thalamus reliability while a notably low level of stimulation current is administered in comparison with the cDBS protocol. Particularly, the beta band peaks in the power spectrum density (PSD) of the parkinsonian STN, GPe, and GPi activities were robustly suppressed and shifted to their normal range by aDBS. Comparison between aDBS and cDBS shows that the aDBS protocol with amplitude modulation can be more efficient at different stimulation frequencies, that is, abnormal beta oscillations were effectively suppressed while the administered stimulation current was reduced. Developing such closed-loop models of aDBS may contribute to the pre-clinical testing and clinical optimization of more efficient aDBS techniques by reducing stimulation current to reduce potential side effects in patients with PD undergoing treatment.

## 2. Methods

### 2.1. Network model

**Figure 1:**
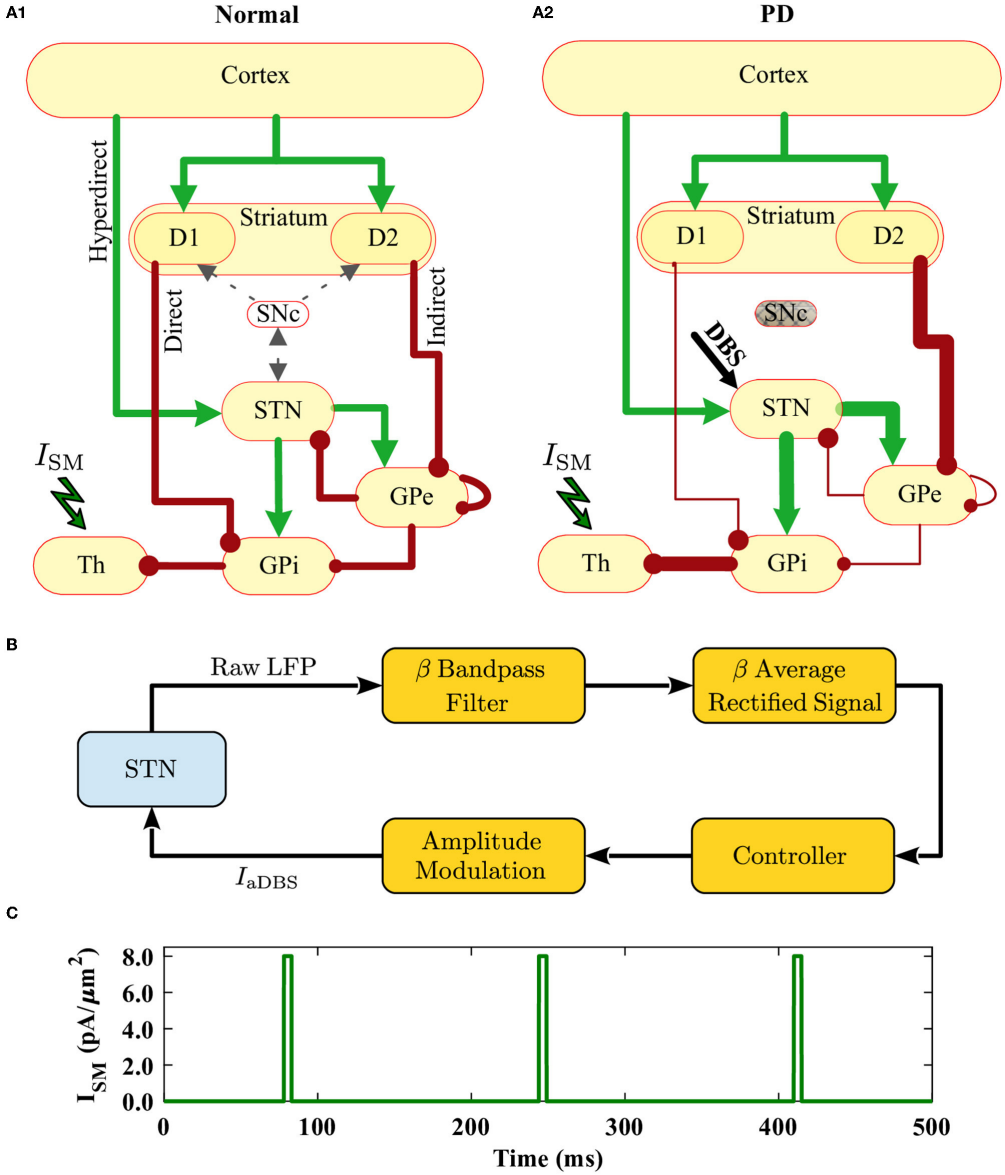
Schematic illustration of the cortico-BG-thalamic network model and closed-loop stimulator. **(A1, A2)** The cortico-BG-thalamic circuitry in the normal **(A1)** and PD **(A2)** conditions. Excitatory (green) and inhibitory (red) pathways are marked by arrows where their relative thickness indicates the strength of the input. STN was the target of DBS in the PD condition. **(B)** Schematic representation of the closed-loop stimulator utilizing STN beta band activity. The raw LFP recorded from STN is beta band (15–30 Hz) filtered, rectified, and averaged to calculate the average rectified value (ARV) of the LFP beta band activity. The beta ARV is then fed to the controller, which updates the amplitude of the stimulation current. **(C)** Time trace of the cortical sensorimotor input (*I*_SM_) to the thalamus given by Equation (18) below.

We considered a bio-inspired and comprehensive cortico-BG-thalamic network model implemented in MATLAB comprising cortex (simulated as 500 external inputs), striatal D1 MSNs (85 neurons) and D2 MSNs (85 neurons), GPe (17 neurons), GPi (17 neurons), STN (137 neurons), and thalamus (140 neurons), as schematically shown in Figure 1A1. The ratio of cells was estimated based on the experimentally reported number of neurons per volume, that is, neuronal density in rats (Oorschot, 1996). Connection probability and the strength of synaptic connections between different pathways used in our simulations are shown in Table 1, which are chosen in accordance with experimental observations in rats (Kita and Kita, 1994; Mink, 1996; Baufreton et al., 2009). Specifically, in the PD state, *D*2 → *GPe* synaptic strength was increased, whereas *D*1 → *GPi* and *GPe* → *GPe* synaptic strengths were decreased with respect to the normal state (see Figures 1A1, A2). Furthermore, an external current mimicking the input from other brain regions was applied to STN, GPe, and GPi, that is, $I_{\text{app}}\left(STN\right)=18pA/\mu m^{2}$, $I_{\text{app}}\left(GPe\right)=12pA/\mu m^{2}$, and $I_{\text{app}}\left(GPi\right)=4.0pA/\mu m^{2}$ in normal condition, and $I_{\text{app}}\left(STN\right)=15.5pA/\mu m^{2}$, $I_{\text{app}}\left(GPe\right)=0.4pA/\mu m^{2}$, and $I_{\text{app}}\left(GPi\right)=0.0pA/\mu m^{2}$ in the PD state. Other parameters in the PD state were similar to those used in the normal state.

**Table 1:** Connection probability and the strength of synaptic connections between different pathways used in our simulations in normal and PD conditions (Mink, 1996; Leblois et al., 2006; Corbit et al., 2016).

**Pathway**	**Connection probability**	***g***(***nS***/***μm***^**2**^)	
		**Normal**	**PD**
*STN* → *GPe*	40%	0.82	0.82
*STN* → *GPi*	40%	0.15	0.15
*GPe* → *STN*	7%	0.14	0.14
*GPe* → *GPi*	6%	1.39	1.39
*GPe* → *GPe*	45%	0.61	0.25
*D*1 → *GPi*	37.5%	0.225	0.08
*D*2 → *GPe*	37.5%	0.221	0.66
*GPi* → *Th*	70%	0.03	0.03

In the cortico-BG-thalamic circuitry shown in Figure 1A1, the striatum receives excitatory inputs from the cortex and relays them toward GPi using two competing pathways: the direct pathway comprising striatal D1 receptor expressing MSNs and the indirect pathway governed by D2 receptor expressing MSNs. Cortical inputs in the indirect pathway are then mediated by the inhibitory GPe neurons, which are bidirectionally connected to STN neurons. The output of STN is then transmitted to the GPi, which inhibits the thalamus. The thalamus sends excitatory input to the cortex and receives excitatory feedback. The STN also receives direct excitatory input from the cortex using the hyperdirect pathway. Altered synaptic transmission in the PD condition with respect to normal condition is schematically presented in Figures 1A1, A2, by changing the relative thickness of arrows in different pathways.

### 2.2. Neuron model

#### 2.2.1. STN neurons

The membrane potential dynamics of STN neurons are described by a single-compartment conductance-based model introduced by Terman et al. (2002), as follows:


(1) $$\begin{array}{l} C_{\text{m}}\frac{dV_{\text{i}}}{dt}=-I_{\text{L}}-I_{\text{K}}-I_{\text{Na}}-I_{\text{T}}-I_{\text{Ca}}-I_{\text{AHP}}-I_{\text{GPe}\to \text{STN}}+I_{\text{SM}}+I_{\text{app}}+I_{\text{DBS}}, \end{array}$$

where $C_{\text{m}}=1pF/\mu m^{2}$ is the membrane capacitance. *I*_GPe → STN_ is the synaptic current, *I*_SM_ is the cortical sensorimotor input to the thalamus, *I*_app_ is the external applied current, and *I*_DBS_ is the stimulation current (see below). The leak current (*I*_L_), potassium current (*I*_K_), sodium current (*I*_Na_), high-threshold calcium current (*I*_Ca_), and calcium-dependent afterhyperpolarization (AHP) (*I*_AHP_) are described by Hodgkin–Huxley type equations as follows:


(2) $$\begin{array}{l} \begin{array}{l} I_{\text{L}}\left(V\right)=g_{\text{L}}\left(V-V_{\text{L}}\right),\\ I_{\text{K}}\left(V\right)=g_{\text{K}}n^{4}\left(V-V_{\text{K}}\right),\\ I_{\text{Na}}\left(V\right)=g_{\text{Na}}m_{\infty }^{3}\left(V\right)h\left(V-V_{\text{Na}}\right),\\ I_{\text{Ca}}\left(V\right)=g_{\text{Ca}}s_{\infty }^{2}\left(V\right)\left(V-V_{\text{Ca}}\right),\\ I_{\text{T}}\left(V\right)=g_{\text{T}}a_{\infty }^{3}\left(V\right)b_{\infty }^{2}\left(r\right)\left(V-V_{\text{Ca}}\right),\\ I_{\text{AHP}}\left(V\right)=g_{\text{AHP}}\left(V-V_{\text{K}}\right)\left(\left[\text{Ca}\right]/\left(\left[\text{Ca}\right]+k_{1}\right)\right). \end{array} \end{array}$$

The slowly operating gating variables (*X* = *n, h, r*) are treated as functions of both time and voltage and have first-order kinetics governed by differential equations of the form:


(3) $$\begin{array}{l} \begin{array}{l} dX/dt=\phi_{\text{X}}\left(\left(X_{\infty }\left(V\right)-X\right)/\tau_{\text{X}}\left(V\right)\right),\\ \tau_{\text{X}}\left(V\right)=\tau_{X}^{0}+\tau_{X}^{1}/\left(1+\exp\left(-\left(V-\theta_{\text{X}}^{\tau }\right)/\sigma_{\text{X}}^{\tau }\right)\right), \end{array} \end{array}$$

where activation (and inactivation) time constants have a sigmoidal dependence on voltage, such that the voltage at which the time constant is midway between its maximum and minimum values is θ^τ^, and σ^τ^ is the slope factor for the voltage dependence of the time constant (see **Table 3**).

Activation gating for the rapidly activating channels (*m*, *a*, and *s*) was treated as instantaneous. For all gating variables (*X* = *n, m, h, a, r, s*), the steady-state voltage dependence was determined using:


(4) $$\begin{array}{l} \begin{array}{l} X_{\infty }\left(V\right)={\left[1+\exp\left(-\frac{V-V_{\text{X}}}{k_{\text{X}}}\right)\right]}^{-1},\\ I_{\text{T}}:b_{\infty }\left(r\right)={\left[1+\exp\left(\left(r-\theta_{\text{b}}\right)/\sigma_{\text{b}}\right)\right]}^{-1}-{\left[1+\exp\left(-\theta_{\text{b}}/\sigma_{\text{b}}\right)\right]}^{-1}. \end{array} \end{array}$$

The intracellular concentration of *Ca*^2+^ ions ([Ca]) is governed by the differential equation *d*[Ca]/*dt* = ε(−*I*_Ca_ − *I*_T_ − *k*_Ca_[*Ca*]). The constant ε combines the effects of buffers, cell volume, and the molar charge of calcium in units of mole-s/coulombs-liter. The constant *k*_1_ is the dissociation constant of the calcium-dependent AHP current. The constant *k*_Ca_ is the calcium pump rate constant in units of coulombs-liter/mole-s. Relevant kinetic parameters used in simulations are presented in Tables 2, 3.

**Table 2:** Kinetic parameters for STN, GP (GPe/GPi), and thalamus.

**Variable**	**Nucleus**	**θ_*x*_**	**σ_*x*_**	** $\tau_{x}^{0}$ **	** $\tau_{x}^{1}$ **	** $\theta_{x}^{\tau }$ **	** $\sigma_{x}^{\tau }$ **	***Q*_*x*_**
*m*	STN	–30	15	—	—	—	—	—
	GP	–37	10	—	—	—	—	—
	Th	–37	7	—	—	—	—	—
*h*	STN	–39	–3.1	1	500	–57	–3	0.75
	GP	–58	–12	0.05	0.27	–40	-12	0.05
	Th	–41	4	—	—	—	—	—
*n*	STN	–32	8	1	100	–80	–26	0.75
	GP	–50	14	0.05	0.27	-40	–12	0.1
*r*	STN	–67	–2	7.1	17.5	68	–2.2	0.5
	GP	–70	–2	30	0	—	—	1
	Th	–84	4	—	—	—	—	—
*a*	STN	–63	7.8	—	—	—	—	—
	GP	–57	2	—	—	—	—	—
*s*	STN	–39	8	—	—	—	—	—
	GP	–35	2	—	—	—	—	—
*b*	STN	0.4	–0.1	—	—	—	—	—
*p*	Th	–60	6.2	—	—	—	—	—

**Table 3:** Maximal conductances (*g*_x_), calcium dynamic parameters, and reversal potentials (*E*_x_) of the membrane currents for STN, GP (GPe/GPi), and thalamus.

	$\bar{g}$ **(*****mS***/***cm***^**2**^**)**									**E (mV)**			
	**L**	**K**	**Na**	**T**	**Ca**	**AHP**	**ε_Ca_**	***k*_Ca_**	***k*_1_**	**L**	**K**	**Na**	**Ca**
STN	2.25	45	37.5	0.5	0.5	9	3.75 × 10^−5^	22.5	15	–60	–80	55	140
GP	0.1	30	120	0.5	0.1	30	1.00 × 10^−4^	20	30	–55	–80	55	120
Th	0.05	5	3	5	—	—	—	—	—	–70	–90	50	0

#### 2.2.2. GPe/GPi neurons

The membrane potential dynamics of GPe neurons are described as follows Terman et al. (2002) and Rubin and Terman (2004):


(5) $$\begin{array}{c} \begin{array}{l} C_{\text{m}}\frac{dV_{\text{i}}}{dt}=-I_{\text{L}}-I_{\text{K}}-I_{\text{Na}}-I_{\text{T}}-I_{\text{Ca}}-I_{\text{AHP}}\\ \;-I_{\text{STN}\to \text{GPe}}-I_{\text{GPe}\to \text{GPe}}-I_{\text{D}2\to \text{GPe}}+I_{\text{app}}. \end{array} \end{array}$$

The ionic currents are similar to STN neurons, as described in Equation (2) except for the low-threshold T-type calcium current (*I*_T_) that is defined differently:


(6) $$\begin{array}{c} I_{\text{T}}\left(V\right)=g_{\text{T}}a_{\infty }^{3}\left(V\right)r\left(V-V_{\text{Ca}}\right), \end{array}$$

where the dynamics of gating variable *a* are similar to Equation (4) and the dynamics of variable *r* are the same as Equation (3). GPe parameters used in simulations are presented in Tables 2, 3.

The dynamics of GPi neurons were modeled similar to the dynamics of GPe neurons. We used the following current balance equation to calculate the GPi membrane potential:


(7) $$\begin{array}{c} \begin{array}{l} C_{\text{m}}\frac{dV_{\text{i}}}{dt}=-I_{\text{L}}-I_{\text{K}}-I_{\text{Na}}-I_{\text{T}}-I_{\text{Ca}}-I_{\text{AHP}}\\ -I_{\text{STN}\to \text{GPi}}-I_{\text{GPe}\to \text{GPi}}-I_{\text{D}1\to \text{GPi}}+I_{\text{app}}. \end{array} \end{array}$$

The corresponding numerical values for parameters are shown in Tables 2, 3.

#### 2.2.3. Thalamic neurons

The membrane potential dynamics of thalamic cells are modeled as follows Rubin and Terman (2004):


(8) $$\begin{array}{c} \begin{array}{l} C_{\text{m}}\frac{dV_{\text{Th}}}{dt}=-I_{\text{L}}-I_{\text{K}}-I_{\text{Na}}-I_{\text{T}}-I_{\text{GPi}\to \text{Th}}+I_{\text{SM}}. \end{array} \end{array}$$

The ionic currents *I*_Na_ and *I*_L_ are similar to those defined for the STN neurons, as described in Equation (2), whereas *I*_T_ and *I*_K_ are defined as follows:


(9) $$\begin{array}{c} \begin{array}{l} I_{\text{K}}\left(V\right)=g_{\text{K}}{\left[0.75\left(1-h_{\text{Th}}\right)\right]}^{4}\left(V-V_{\text{K}}\right),\\ I_{\text{T}}\left(V\right)=g_{\text{T}}p_{\infty }^{2}\left(V\right)r\left(V-V_{\text{T}}\right). \end{array} \end{array}$$

The gating variables are of the form:


(10) $$\begin{array}{c} \begin{array}{l} dh\left(t\right)/dt=\left(h_{\infty }\left(V_{\text{Th}}\right)-h_{\text{Th}}\right)/\tau_{h}\left(V_{\text{Th}}\right),\\ dr\left(t\right)/dt=\left(r_{\infty }\left(V_{\text{Th}}\right)-r_{\text{Th}}\right)/\tau_{r}\left(V_{\text{Th}}\right),\\ \tau_{\text{h}}\left(V\right)=1/\left(a_{h}+b_{h}\right),\\ a_{h}=0.128\exp\left(-\left(V+46\right)/18\right),\\ b_{h}\left(V\right)=4/\left[1+\exp\left(-\left(V+23\right)/5\right)\right],\\ \tau_{r}\left(V\right)=0.4\left[28+\exp\left(-\left(V+25\right)/10.5\right)\right]. \end{array} \end{array}$$

Relevant kinetic parameters used in simulations are presented in Table 2.

#### 2.2.4. Striatum: D1 and D2 MSNs

Two subpopulations of neurons representing D1 and D2 receptor-expressing MSNs were considered to model the striatum. The membrane potential dynamics for MSNs are of the form (Mahon et al., 2000):


(11) $$\begin{array}{c} C_{\text{m}}\frac{dV_{\text{i}}}{dt}=-I_{\text{L}}-I_{\text{K}}-I_{\text{Na}}-I_{\text{Kir}}-I_{\text{Af}}-I_{\text{As}}-I_{\text{Krp}}-I_{\text{NaP}}-I_{\text{NaS}}. \end{array}$$

The ionic currents (*I*_Na_, *I*_K_, and *I*_L_) are similar to those used for modeling the STN neurons, as described in Equation (2), but gating variables were taken from the study of Wang and Buzsáki (1996). The gating variable *m* was approximated by *m*_∞_ = α_m_/(α_m_ + β_m_), where α_m_(*V*) = −0.1(*V* + 35)/(exp(−0.1(*V* + 35)) − 1) and β_m_(*V*) = 4 exp(−(*V* + 60)/18). Other gating variables (*X* = *h, n*) obey the following first-order kinetics:


(12) $$\begin{array}{c} dX/dt=\phi \left(\alpha_{X}\left(1-X\right)-\beta_{X}X\right) \end{array}$$

where ϕ is constant, α_*h*_(*V*) = 0.07 exp(−(*V* + 58)/20), β_*h*_(*V*) = 1/(exp(−0.1(*V* + 28)) + 1), α_*n*_(*V*) = −0.01(*V* + 34)/(exp(−0.1(*V* + 34)) − 1), and β_*n*_(*V*) = 0.125 exp(−(*V* + 44)/80).

Fast (*I*_Af_) and slow (*I*_As_) A-type potassium currents, inward rectifier potassium current (*I*_Kir_), persistent potassium current (*I*_Krp_), and persistent (*I*_NaP_) and slowly inactivating (*I*_NaS_) sodium currents are defined as follows (Wood et al., 2004):


(13) $$\begin{array}{c} \begin{array}{l} I_{X}\left(V\right)=g_{X}m_{\infty }^{\text{k}}\left(V\right)h\left(V-{\text{E}}_{X}\right), \end{array} \end{array}$$

where *X* ∈ {*Kir, Af, As, Krp, NaS, NaP*}. Gating variables obey differential equations defined in Equations (3), (4). Other parameters are defined as follows:


(14) $$\begin{array}{c} \begin{array}{l} \tau \left(V\right)=\tau_{0}{\left[\exp\left(-\frac{V-V_{\text{τ}}}{k_{\text{τ}}}\right)+\exp\left(\frac{V-V_{\text{τ}}}{k_{\text{τ}}}\right)\right]}^{-1}, \end{array} \end{array}$$

except for the inactivation of slow A-type potassium current for which the kinetics were defined by $\tau_{\text{hAs}}\left(V\right)=1790+2930\cdot \exp\left(-{\left(\frac{V+38.2}{28}\right)}^{2}\right)\cdot \left(\frac{V+38.2}{28}\right)$. The numerical values of parameters used in our simulations are listed in Table 4.

**Table 4:** Model parameters for striatal MSNs.

			***X***_**∞**_(***V***)			τ(***V***)		
**Current**	***m*^k^, *h***	** $\bar{g}\left(mS/cm^{2}\right)$ **	***V*_x_(*mV*)**	***k*_x_(*mV*)**	**E(*mV*)**	**τ_0_(*ms*)**	***V*_τ_(*mV*)**	***k*_τ_(*mV*)**
Kir	*m*_Kir_	0.15	–100	–10	–90	—	—	—
Af	*m*_Af_	0.09	–33.1	7.5	–73	1	—	—
	*h*_Af_		–70.4	–7.6		25	—	—
As	*m*_As_	0.32	–25.6	13.3	–85	131.4	–37.4	27.3
	*h*_As_		–78.8	—10.4		—	—	—
Krp	*m*_Krp_	0.42	–13.4	12.1	–77.5	206	–53.9	26.5
	*h*_Krp_		–55	–19		—	—	—
NaP	*m*_NaP_	0.02	–47.8	3.1	45	1	—	—
NaS	*m*_NaS_	0.11	–16	9.4	40	637.8	–33.5	26.3

#### 2.2.5. Synaptic currents

The synaptic current *I*_α → β_ from the presynaptic nucleus (α) to the postsynaptic nucleus (β), with α ∈ {*STN, GPe, GPi, D*1, *D*2}, and β ∈ {*STN, GPe, GPi, Th*}, is given by (Rubin and Terman, 2004):


(15) $$\begin{array}{c} I_{\alpha \to \beta }=g_{\alpha \to \beta }\left(V_{\text{α}}-{\text{E}}_{\alpha \to \beta }\right)\underset{\alpha }{\sum }s_{\alpha }\left(t\right), \end{array}$$

where *g*_α → β_ is the maximal synaptic conductance presented in Table 1, and E_α → β_ is the synaptic reversal potential presented in Table 3. *s*_α_(*t*) represents the synaptic gating variable that obeys the following differential Equation (Rubin and Terman, 2004):


(16) $$\begin{array}{c} \frac{ds_{\text{α}}}{dt}=A_{\text{α}}\left(1-s_{\text{α}}\right)\cdot H_{\text{∞}}\left(V_{\text{α}}-\theta_{\text{α}}\right)-B_{\text{α}}s_{\text{α}}, \end{array}$$

where $H_{\infty }\left(V_{\alpha }\right)=1/\left(1+\exp\left[-\left(V_{\alpha }-\theta_{\alpha }^{H}\right)/\sigma_{\alpha }^{H}\right]\right)$ is a smooth approximation of the Heaviside step function (relevant parameters are given in Table 5), and *A*_α_ and *B*_α_ control the synaptic time courses.

**Table 5:** Model parameters of the smooth approximation of the Heaviside step function for STN, GP (GPe/GPi), and D1/D2 MSN.

	** $\theta_{\alpha }^{H}$ **	** $\sigma_{\alpha }^{H}$ **	**θ_α_**
STN	–39.0	8.0	30.0
GP	–57.0	2.0	20.0
MSN	–42.0	5.0	18.0

#### 2.2.6. Cortical current

The cortical sensorimotor input to the thalamus is approximated as a train of rectangular depolarizing current pulses (*I*_SM_), which is shown in Figure 1C, based on Equation (18) (Rubin and Terman, 2004):


(17) $$\begin{array}{c} I_{\text{SM}}=i_{\text{SM}}\text{H}\left(\sin\left(2\pi t/\rho_{\text{SM}}\right)\right)\cdot \left[1-\sin\left(\frac{2\pi \left(t+\delta_{\text{SM}}\right)}{\rho_{\text{SM}}}\right)\right], \end{array}$$

Where $i_{\text{SM}}=8pA/\mu m^{2}$ is the amplitude of the current, ρ_SM_ = 166*ms* denotes the period of the current signal, and δ_SM_ = 5*ms* represents the duration of each individual pulse.

### 2.3. Stimulation protocol

The stimulation was administered to the STN as schematically shown in Figure 1A2. The stimulation current was modeled by the following protocol (Rubin and Terman, 2004):


(18) $$\begin{array}{c} I_{\text{DBS}}=i_{\text{DBS}}\text{H}\left(\sin\left(2\pi t/\rho_{\text{DBS}}\right)\right)\cdot \left[1-\sin\left(\frac{2\pi \left(t+\delta_{\text{DBS}}\right)}{\rho_{\text{DBS}}}\right)\right], \end{array}$$

Where $i_{\text{DBS}}=2mA/\mu m^{2}$ is the amplitude of the stimulation signal, ρ_DBS_ = 1/130*ms* denotes the stimulation period, and δ_DBS_ = 5*ms* is the duration of individual stimulation pulses (Fleming et al., 2020a). In the cDBS protocol, the model stimulation signal was continuously delivered to the STN with a 130-Hz frequency (Fleming et al., 2020a). The same frequency was used for the aDBS protocol; however, the amplitude of the signal was modulated based on a closed-loop control scheme described later.

### 2.4. Data analysis

The LFP of the oscillatory neural activity was defined as $LFP\left(t\right)=N^{-1}\underset{\alpha }{\sum }s_{\alpha }\left(t\right)$, where *s*(*t*) is the synaptic variable introduced in Equation (17). Rigorous computational approximations showed that a simple weighted sum of the model synaptic currents excellently captures the time course of the LFP signal (Mazzoni et al., 2015). This provides a simple formula by which the LFP signal can be estimated directly from network activity, providing a missing quantitative link between simplified neuronal models and LFP measures *in vivo* (Mazzoni et al., 2015).

The beta band-filtered LFP of the STN was calculated by using the bandpass filter of the simulated raw STN LFP using the bandpass filter function implemented in MATLAB within the frequency range of 15–30 Hz.

The power spectrum of each calculated signal was computed by the fast Fourier transform (FFT) function implemented in MATLAB.

### 2.5. Closed-loop control scheme

In the closed-loop control of aDBS administered to the STN, the stimulation current is delivered in the form of high-frequency pulses with the same frequency used in the open-loop cDBS but with a modified amplitude. Amplitude modulation was implemented by the closed-loop feedback of the measured beta band LFP activity of the STN, which is schematically shown in Figure 1B. The average rectified value (ARV) of the STN beta band LFP was calculated by full-wave rectifying of the filtered LFP signal. The maximum value of beta ARV in the normal state was assumed as a target value for the beta ARV. During controller simulations, a beta ARV above the target value was considered as the pathological beta activity, while a beta ARV below the target value was assumed as the fluctuations of normal beta activity.

The controller input (*e*) at a given time was calculated as the normalized error between the measured beta ARV (β_measured_) and the target beta ARV (β_target_), which is as follows (Fleming et al., 2020a,b):


(19) $$\begin{array}{c} e\left(t\right)=\frac{\beta_{\text{measured}}\left(t\right)-\beta_{\text{target}}}{\beta_{\text{target}}} \end{array}$$

The controller operated with a sampling interval *T*_s_ = 50*ms* (Fleming et al., 2020a), updating the modulated aDBS parameter at each controller call. Other choices for the sampling time window resulted in the same observed beta power and stimulation performance (see Supplementary Figure S1). The P controller for closed-loop control of the aDBS amplitude can be defined as follows (Fleming et al., 2020a,b):


(20) $$\begin{array}{c} u\left(t\right)=K_{\text{p}}\cdot e\left(t\right). \end{array}$$

where *u*(*t*) is the modulated aDBS parameter value, that is, the stimulation amplitude at a given time, *K*_p_ = 5 (Fleming et al., 2020a) is the controller proportional gain of the aDBS parameter at each controller call, and *e*(*t*) is the controller error input signal at a given time. The aDBS current is given as follows:


(21) $$\begin{array}{c} I_{\text{aDBS}}\left(t\right)=u\left(t\right)\cdot I_{\text{DBS}}\left(t\right). \end{array}$$

### 2.6. Stimulation performance assessment

Computational results show that synchronized activity interrupts the thalamic reliability to transmit sensorimotor inputs, which may lead to akinesia and rigidity (Rubin and Terman, 2004). One way to assess and compare the efficiency of different DBS protocols in restoring sensorimotor functionality is their effectiveness in improving the response of the thalamus to sensorimotor stimuli. Thalamic reliability (R) is a measure that quantifies the faithfulness of the thalamic relay defined in terms of the generation of thalamo-cortical activity patterns that match the inputs to thalamo-cortical cells. It is determined by the fraction of sensorimotor stimuli that elicit a single action potential in the thalamus so that a *missed* spike is recorded when no spikes are fired in response to a sensorimotor input, whereas a *bad* spike is recorded when multiple spikes are fired in response to a single sensorimotor input. The reliability of transmitting information of the thalamus can be regarded as an evaluation of the effectiveness of DBS. This is quantified by the error index introduced by Rubin and Terman (2004) for the fidelity of thalamic throughput such that the minimal error is achieved when each sensorimotor input pulse results in a single action potential in a thalamic neuron (also see Supplementary Figure S2 and Section 4). The reliability of the thalamus is defined as follows (Gorzelic et al., 2013):


(22) $$\begin{array}{c} R=1-\frac{b+m}{N_{\text{SM}}}. \end{array}$$

where *b* is the number of bad spikes, *m* is the number of missed spikes, and *N*_SM_ is the total number of sensorimotor inputs in the simulation.

Another way to quantify the performance of stimulation is to calculate the energy (power) expenditure index (E), which is a measure of the amount of administered stimulation current, defined as the root mean square (RMS) of the stimulation current signal (Su et al., 2018) as follows:


(23) $$\begin{array}{c} E=\sqrt{\frac{1}{T}\int_{T}I_{\text{DBS}}^{2}dt}. \end{array}$$

where *T* is the total time of the simulation.

Ultimately, the beta suppression efficiency of cDBS and aDBS protocols was quantified as the percentage of beta suppression in the STN per unit of the consumed energy, defined as follows (Fleming et al., 2020a):


(24) $$\begin{array}{c} \eta =\frac{1}{E}\times \left(1-\frac{1}{T}\int_{T}\frac{\beta_{\text{NoDBS}}\left(t\right)-\beta_{\text{DBS}}\left(t\right)}{\beta_{\text{NoDBS}}\left(t\right)}dt\right)\times 100 \end{array}$$

where E was introduced in Equation (24), *T* is the total time of simulation, β_NoDBS_(*t*) is the beta ARV signal measured when DBS is off, and β_DBS_(*t*) is the beta ARV signal measured when DBS was administered.

## 3. Results

### 3.1. Properties of normal and PD network model

**Figure 2:**
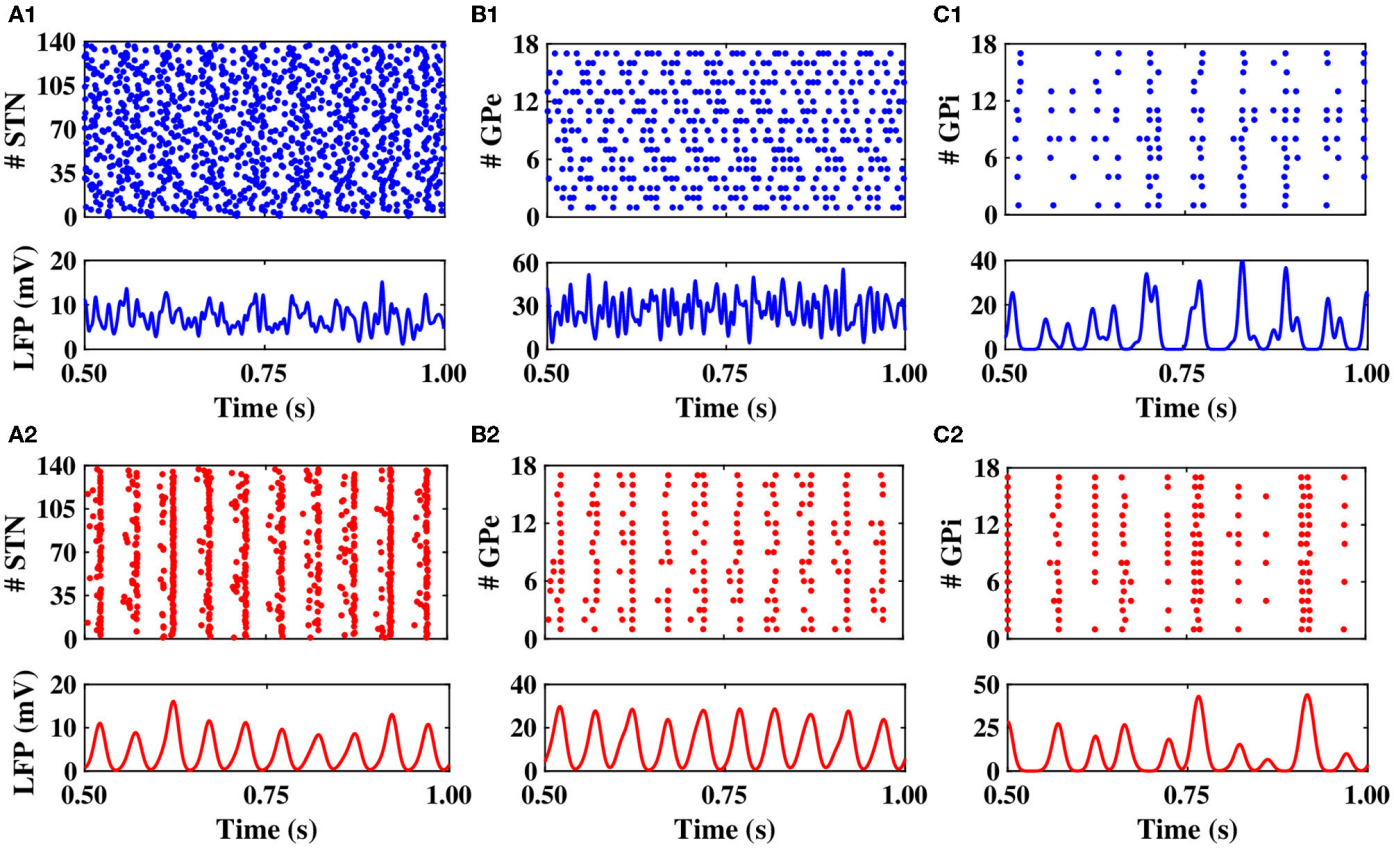
Population dynamics of the STN, GPe, and GPi in normal and PD conditions. Raster plots (top) and LFPs (bottom) of the STN, GPe, and GPi activities in normal **(A1–C1)** and PD **(A2–C2)** conditions.

First, we set the model parameters to mimic the normal and PD network dynamics. The raster plots shown in Figures 2A1–C1, top illustrate the dynamics of STN, GPe, and GPi neurons in normal condition, respectively. The synchronized neural activity led to pronounced rhythmic activity and large-amplitude oscillations in the LFP of different nuclei (shown in Figures 2A1–C1, bottom). The raster plots and LFP of STN, GPe, and GPi neurons in the PD condition are shown in Figures 2A2–C2.

**Figure 3:**
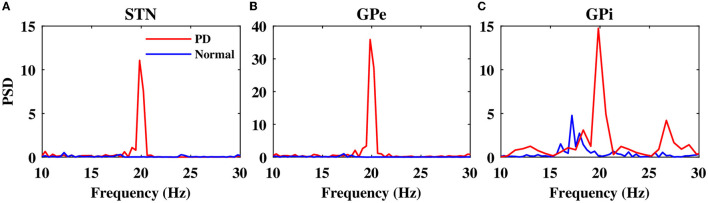
Power spectrum of the STN, GPe, and GPi LFP activities. Power spectrum density of the STN **(A)**, GPe **(B)**, and GPi **(C)** LFP activities in normal (blue) and PD (red) conditions.

Notably, in the normal condition, STN exhibited a relatively desynchronized neural activity (see Figure 2A1, top), characterized by small-amplitude oscillations in the STN LFP shown in Figure 2A1, bottom. In the PD state, however, the activity of STN neurons became strongly synchronized (Figure 2A2, top), characterized by large-amplitude rhythmic oscillations in the STN LFP (Figure 2A2, bottom). The mean firing rate of STN neurons in the normal state was 12 ± 0.6*Hz*, which increased to 19 ± 0.8*Hz* in the PD state. The PSD of STN activity in the PD state is characterized by a sharp peak in the beta band (approximately 20 Hz) as shown in Figure 3A (red), whereas the normal PSD hardly showed any pronounced peak (Figure 3A, blue).

**Figure 4:**
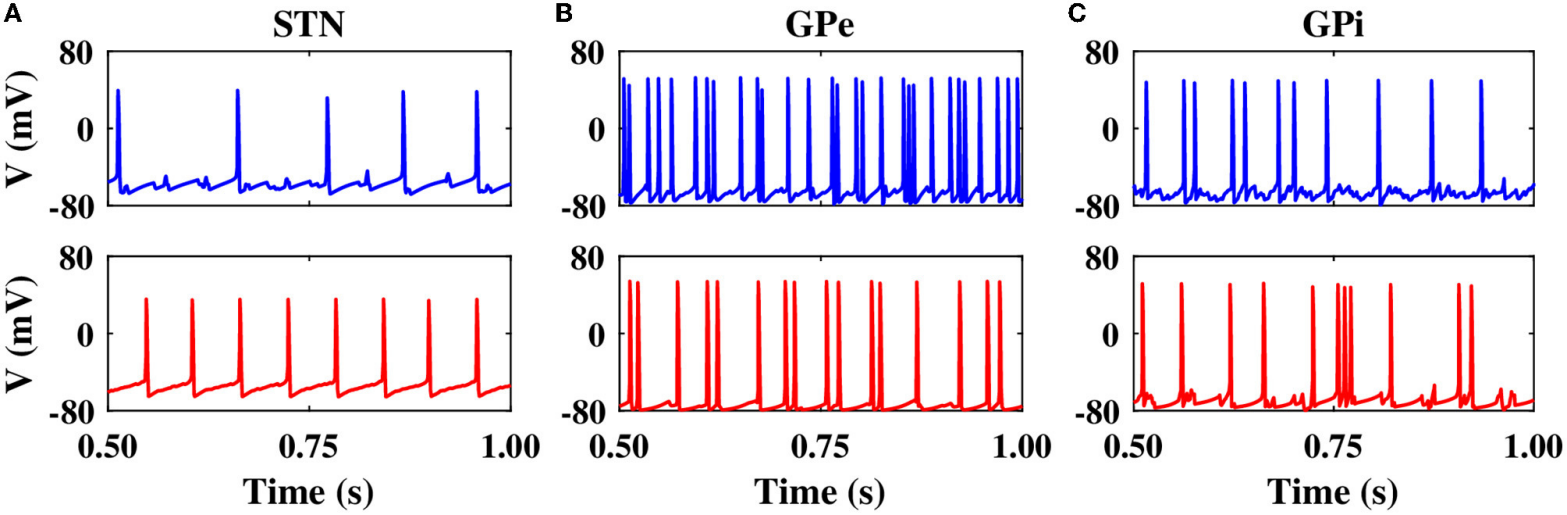
Single-cell membrane voltage traces of STN, GPe, and GPi neurons. Time course of membrane voltages of single STN **(A)**, GPe **(B)**, and GPi **(C)** neurons randomly chosen from the network in normal (top) and PD (bottom) conditions.

In the normal condition, GPe neurons fired in a relatively irregular manner, as it is shown in the raster plot (Figure 2B1, top) and LFP activity (Figure 2B1, bottom), with a mean firing rate of 60 ± 2.4*Hz*. In the PD state, the mean firing rate of GPe neurons decreased to 32 ± 1.3*Hz* where the firing activity of neurons was more synchronized (Figure 2B2, top), characterized by rhythmic LFP oscillations shown in Figure 2B2, bottom. The firing activity of GPi neurons, however, was relatively sparse in the normal condition (Figure 2C1), with a mean firing rate of 20 ± 0.9*Hz*. The activity of GPi neurons in the PD condition is shown in Figure 2C2, where the mean firing rate increased to 28 ± 1.3*Hz*. The PSD of GPe and GPi activities in the PD state showed a sharp peak at approximately 20 Hz (Figures 3B, C, red), whereas their normal PSD did not show any pronounced peak in the beta band (Figures 3B, C, blue). For example, single-cell membrane voltage traces of randomly chosen STN, GPe, and GPi neurons in normal (top) and PD (bottom) conditions are presented in Figure 4.

### 3.2. Suppression of pathological oscillations by model DBS

To suppress parkinsonian beta oscillations within the BG nuclei (i.e., to suppress pronounced peaks in the PSD of STN, GPe, and GPi activities in Figure 3, red), the model stimulation was administered to the STN using two different stimulation protocols, that is, cDBS and aDBS. In the cDBS protocol, high-frequency (130 Hz) stimulation pulses are continuously delivered to STN with a fixed amplitude, as described in Equation (19). In the aDBS protocol, stimulation pulses were continuously delivered to STN with the same frequency that was used in cDBS; however, the stimulation amplitude is modulated by a control signal that sets the current amplitude based on the beta band activity of the STN, as described in Equation (22).

**Figure 5:**
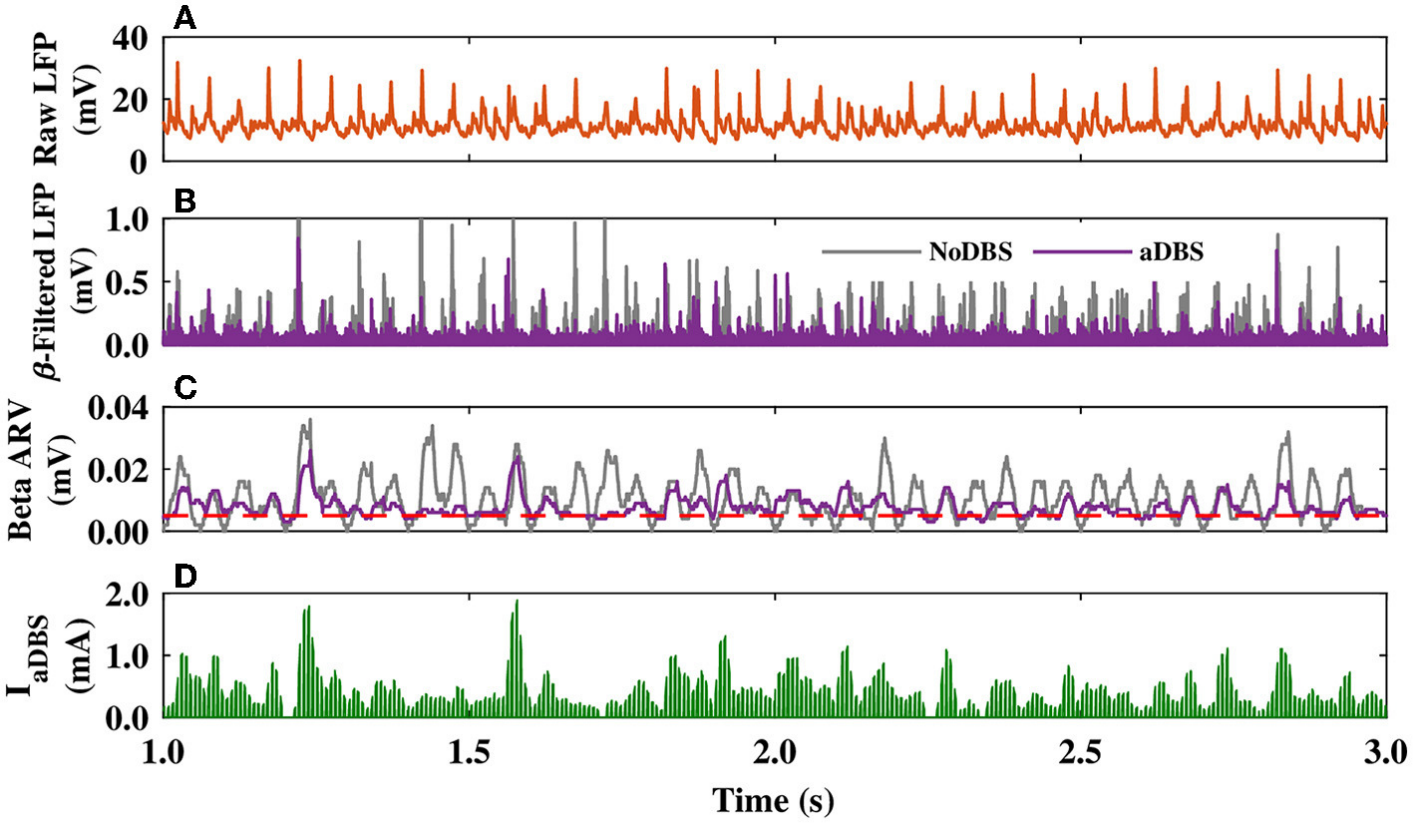
STN LFP and amplitude modulation of the aDBS current. **(A–C)** The simulated raw LFP of the STN **(A)**, beta band filtered LFP **(B)**, and beta ARV **(C)** for DBS off (NoDBS; gray) and aDBS (violet). The target level for the beta ARV (β_target_ = 0.005 mV) is denoted by the red dashed line in **(C)**. **(D)** Amplitude modulation of the aDBS current by the P controller. The aDBS amplitude was restricted between 0.0 and 2.0 mA.

The closed-loop control stimulator of the model aDBS utilizing the STN beta band activity is schematically shown in Figure 1B. In the model, as presented in Figure 5A, the raw LFP recorded from the STN was first filtered in the beta band (15–30 Hz) frequency (Figure 5B, violet); also see Section 2. The beta band filtered output of the parkinsonian STN activity, when the DBS was off (NoDBS), is also depicted for better comparison (Figure 5B, gray). The beta bandpass filtered was then rectified and averaged to calculate the ARV of the LFP beta band activity (Figure 5C). The target level for the beta ARV (i.e., β_target_ = 0.005 mV) is denoted by a red dashed line in Figure 5C, which was estimated based on the STN beta band activity in the normal condition. To efficiently suppress the pathological beta activity within STN (i.e., beta ARV above the target value), the beta ARV is fed to the controller to update the amplitude of the aDBS current, as shown in Figure 5D.

**Figure 6:**
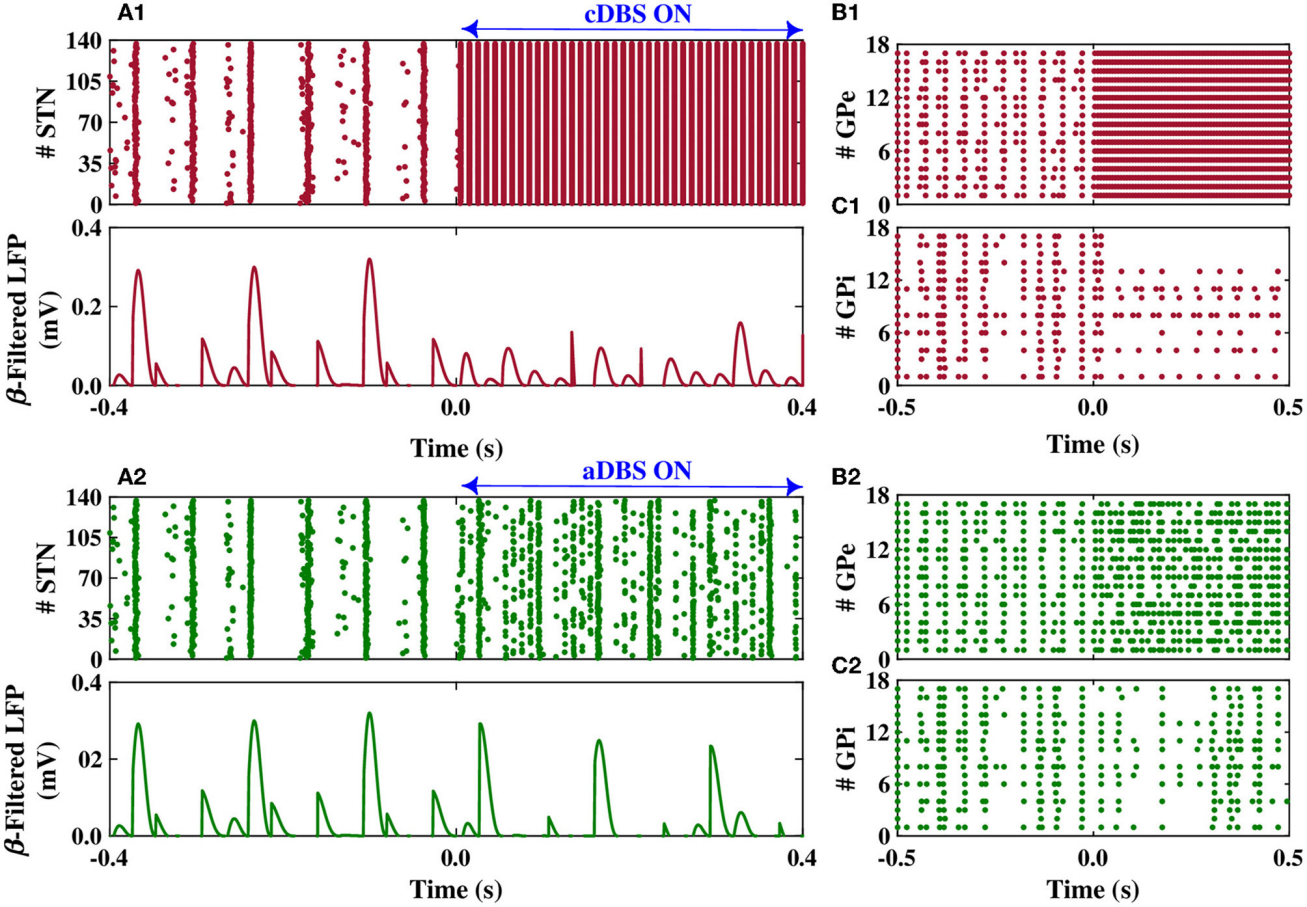
Population dynamics of the STN, GPe, and GPi when the STN was the target of stimulation. Raster plot (top) and beta band filtered LFP (bottom) of the STN, and raster plots of the GPe and GPi when cDBS **(A1–C1)** or aDBS **(A2–C2)** is administered to the STN. The model DBS was off before time *t* = 0 s and was switched on at *t* = 0 s.

The dynamics of STN, GPe, and GPi neurons are shown in Figure 6 when the STN was stimulated by both cDBS and aDBS protocols. Before the stimulation onset (i.e., *t* < 0 s in Figures 6A1, A2), the model parameters were set to mimic the PD state characterized by the overly synchronized neural activity in the STN raster plot (Figures 6A1, A2, top) and by large-amplitude oscillations in the beta band-filtered LFP (Figures 6A1, A2, bottom). The stimulation was then turned on at *t* = 0 s. When cDBS was turned on (i.e., *t* > 0 s in Figure 6A1, top), the activity of STN neurons was entrained to the stimulation frequency (i.e., 130 Hz) and the large-amplitude oscillations in the beta band-filtered LFP were considerably suppressed (Figure 6A1, bottom). When aDBS was used (i.e., *t* > 0 s in Figure 6A2, top), the stimulation pulse train was delivered to the STN with a variable amplitude (see Figure 5D). In this case, the suppression of parkinsonian beta oscillations in the STN was less than cDBS (cf. Figures 6A1, A2, bottom). However, as we will show later, overall less stimulation current was delivered in aDBS while resulting in a more suppression efficiency of the aDBS protocol.

**Figure 7:**
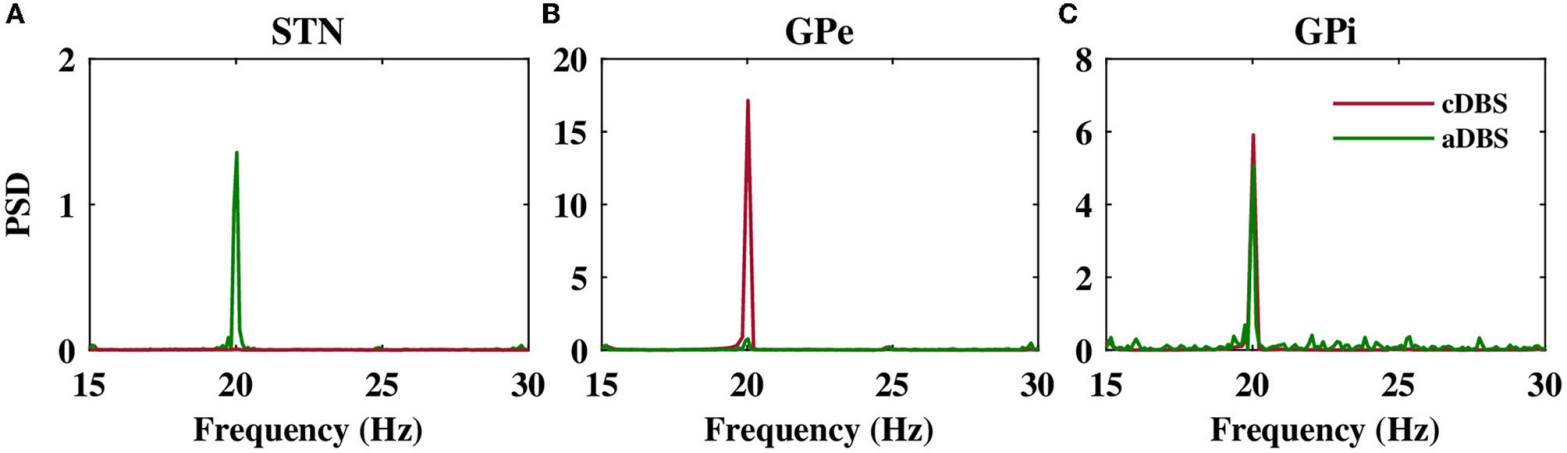
Power spectrum of the STN, GPe, and GPi LFP activities. Power spectrum density of the STN **(A)**, GPe **(B)**, and GPi **(C)** LFP activities when cDBS (maroon) or aDBS (green) is administered to the STN.

The stimulation of the STN not only directly affected the firing activity of STN neurons but also indirectly mediated the firing activity of GPe and GPi neurons. Particularly, cDBS of STN led to the entrainment of GPe neurons to the stimulation frequency (i.e., *t* > 0 s in Figure 6B1), leading to the inhibition of the activity of GPi neurons (i.e., *t* > 0 s in Figure 6C1). On the other hand, aDBS of the STN just increased the firing activity of the GPe neurons and did not result in the entrainment of the GPe activity to the stimulation frequency (i.e., *t* > 0 s in Figure 6B2). Consequently, the activity of GPi neurons was relatively the same before and after stimulation (Figure 6C2). In addition, as it is shown in Figure 7, PSD of the activity of neurons in STN, GPe, and GPi shows that both cDBS and aDBS effectively suppressed beta band oscillations (cf. Figures 3, 7). Interestingly, the suppression of parkinsonian beta oscillations was more pronounced in cDBS of STN (cf. Figure 7A, green and red) and in aDBS of GPe (cf. Figure 7B, green and red). The effects of cDBS and aDBS on the GPi PSD were roughly similar (Figure 7C).

**Figure 8:**
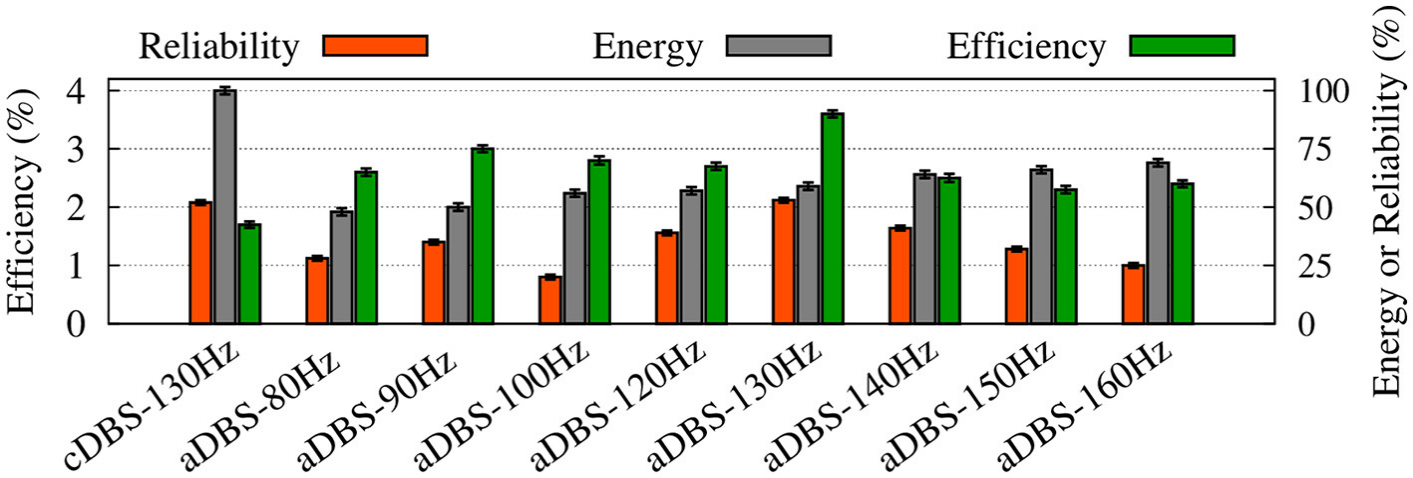
Summary of the performance of cDBS and aDBS protocols. The thalamic reliability (orange), the energy expenditure index as a measure of the amount of delivered stimulation current (gray), and the beta suppression efficiency for the STN (green) of each stimulation protocol at a given frequency were used to assess the performance of stimulation. Standard deviation bars are shown for 10 simulations under each condition.

Differential modulation of the STN, GPe, and GPi beta activities by stimulation was directly related to the model connectivity. While cDBS at 130 Hz effectively suppressed beta activity in the STN, aDBS at the same frequency was less effective in the suppression of STN beta activity, simply because less current was delivered to the STN (Figure 7A). However, we evaluated the stimulation performance based on the percentage of beta suppression in the STN per unit of the consumed energy (see Figure 8). Therefore, based on Figure 8, assuming that the energy consumption of cDBS at 130 Hz was 100%, aDBS at 130 Hz consumed approximately 50% less energy, leading to efficiency about two times as high as the one for cDBS. On the other hand, the STN was connected to the GPe (Table 1; connection strength *g* = 0.82*nS*/μ*m*^2^) more stronger than GPi (Table 1; connection strength *g* = 0.15*nS*/μ*m*^2^). Therefore, cDBS at 130 Hz entrained GPe neurons at the stimulation frequency, leading to an enhanced inhibition among GPe cells and the STN itself, which ultimately prevented effective beta suppression in the GPe. In contrast, adaptive delivery of the stimulation current in the aDBS protocol allowed stimulation to effectively suppress beta activity in the GPe. Finally, weak connections from the STN to GPi minimized the effect of stimulation on GPi, making no particular difference in either case.

### 3.3. Stimulation performance

To evaluate the performance of cDBS vs. aDBS, we calculated thalamic reliability given by Equation (23), the energy expenditure index described in Equation (24) as a measure of the amount of delivered stimulation current, and the beta suppression efficiency of the stimulation protocol defined in Equation (25). The results are presented in Figure 8 where the performance of the cDBS protocol is compared with the aDBS protocol for a variety of stimulation frequencies. The PD condition (NoDBS) was used to set a reference for the thalamic reliability (i.e., 0%). The reference value for the energy expenditure (i.e., delivered stimulation current) was set to 100%, measured when the cDBS protocol (with 130 Hz stimulation frequency) was used for the STN model stimulation. The administration of cDBS led to a 1.7% suppression efficiency and an acceptable value for the thalamic reliability (i.e., 52%).

Interestingly, STN aDBS with the same stimulation frequency as the cDBS protocol (i.e., 130 Hz) led to an increased beta suppression efficiency (i.e., 3.6%), while the energy expenditure was 41% less than cDBS, as shown in Figure 8. Notably, in this case, the value of thalamic reliability was relatively unchanged (i.e., aDBS: 53% vs. cDBS: 52%). Restoring the thalamic reliability and effective suppression of beta oscillations by cDBS comes at the cost of a higher administered stimulation current, resulting in a smaller suppression efficiency than aDBS. In this way, amplitude modulation by closed-loop aDBS (with the same stimulation frequency as the open-loop cDBS) led to more efficient suppression of pathological beta oscillations in the model while notably less stimulation current was used.

As one could expect, increasing the stimulation frequency of aDBS led to increased energy expenditure (Figure 8, gray bars) where the thalamic reliability and suppression efficiency reached their maximum values approximately at 130 Hz stimulation frequency. The overall performance of the stimulation is determined by the trade-off between the energy expenditure and beta suppression outcome of the stimulation protocol.

### 3.4. Monopolar vs. bipolar stimulation

**Figure 9:**
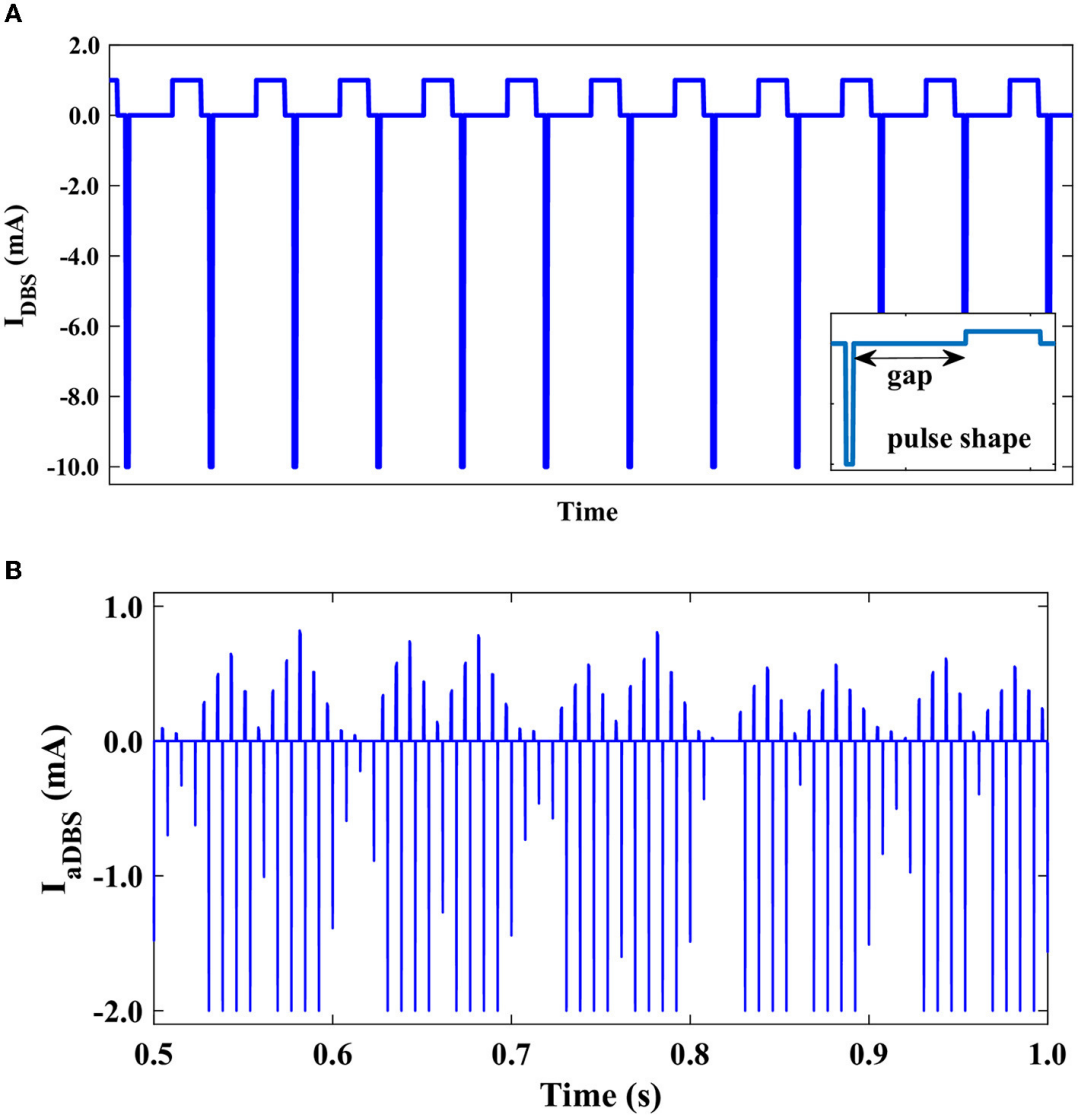
Stimulation signal of electrical biphasic charge-balanced pulses. **(A)** Schematically depicted biphasic charge-balanced pulses without amplitude modulation. Each pulse consists of an interphase gap between the cathodic and anodic phases of the pulse (inset). **(B)** The time course of the bipolar stimulation with amplitude modulation.

Typically, charge-balanced stimuli are used in DBS to avoid tissue damage. We repeated our simulations to test whether the stimulation performance is affected by charge-balanced stimulation. The biphasic charge-balanced stimulation pulses were implemented similar to those used by Popovych and Tass (2019), which consist of a short cathodic pulse (first phase) followed by a longer charge-balancing second phase with opposite polarity. We used the frequency of 130 Hz for the aDBS pulse train and the width of the short pulse (first phase) *PW* = 0.5 ms (Popovych and Tass, 2019). The stimulation signal consisting of electrical biphasic charge-balanced pulses is shown in Figure 9A. The stimulation current can then be constructed as follows (Popovych and Tass, 2019):


(25) $$\begin{array}{c} \begin{array}{ll} I_{\text{DBS}}\left(t\right) & =\{\begin{array}{ll} -10, & t_{n}\leq t<t_{n}+PW,\\ 0, & t_{n}+PW\leq t<t_{n}+PW+GW,\\ 1, & t_{n}+PW+GW\leq t<t_{n}+11PW+GW,\\ 0, & \text{otherwise}, \end{array} \end{array} \end{array}$$

For *t* ∈ (*t*_*n*_, *t*_*n*+1_), where *t*_*n*_ = 1, 000*n*/*f* ms, *n* = 0, 1, 2, … are the times of the pulse onsets, as presented in Figure 9A, and *f* = 130 Hz is the frequency of the stimulation. We considered an interphase time gap of width *GW* = 4.5 ms between the cathodic and anodic phases of the biphasic pulses (Popovych and Tass, 2019). While consistent with previous computational studies (Popovych and Tass, 2019), the interphase gap utilized in our modeling of biphasic stimulation pulses is a fair bit larger than in current DBS systems, where the interphase gap is generally at the smaller time scale of several tens of microseconds (Boogers et al., 2022). This might critically affect the outcome of the biphasic stimulation, for example, shrink the corresponding therapeutic window (Boogers et al., 2022). The amplitude modulation of the bipolar aDBS current is shown in Figure 9B.

**Figure 10:**
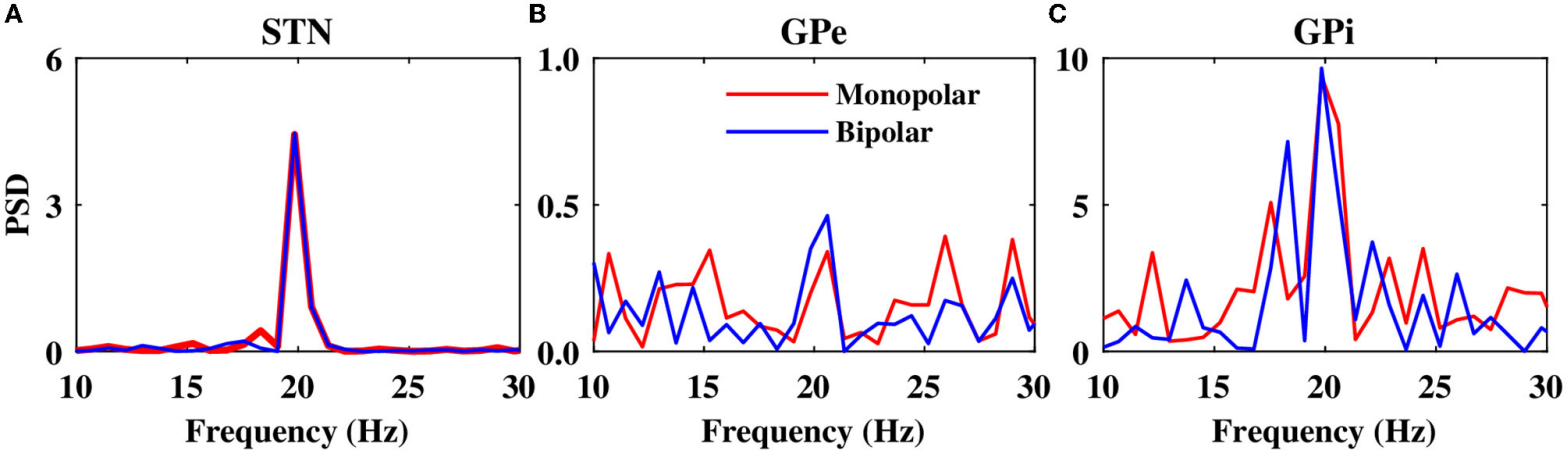
Power spectrum of the LFP activity. Power spectrum density of the STN **(A)**, GPe **(B)**, and GPi **(C)** LFP activities when monopolar aDBS (red) or bipolar aDBS (blue) is administered to the STN.

**Figure 11:**
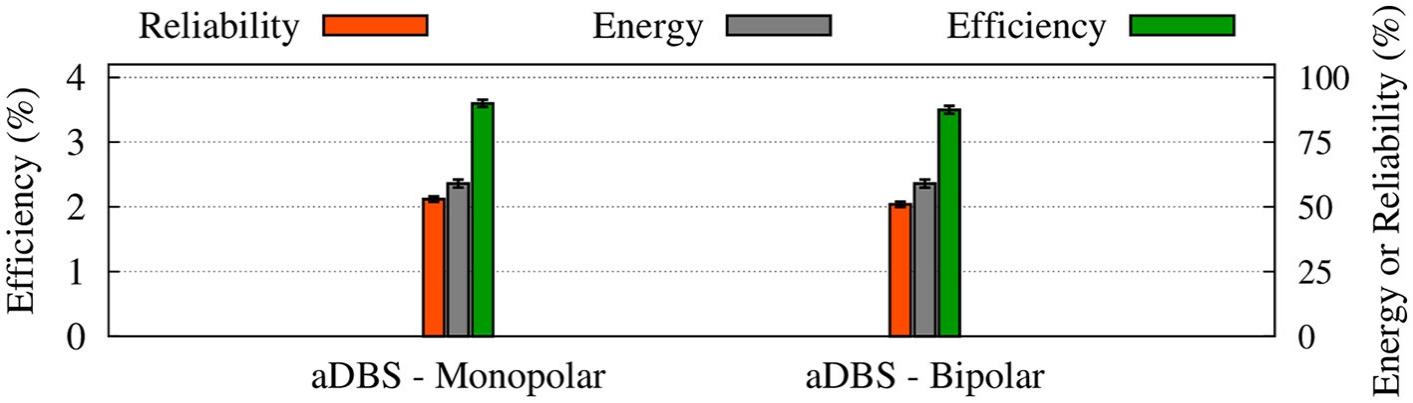
Performance of the monopolar and bipolar aDBS protocols. The thalamic reliability (orange), the energy expenditure index (green), and the beta suppression efficiency for the STN (gray) of each stimulation protocol at 130 Hz frequency were used to assess the performance of stimulation. Standard deviation bars are shown for 10 simulations under each condition.

The power spectrum of the STN, GPe, and GPi LFP activities is shown in Figure 10 when monopolar (red) or bipolar (blue) aDBS is administered to the STN. In addition, the performances of monopolar and bipolar aDBS protocols are presented in Figure 11. Taken together, the results demonstrate that the performance of the model aDBS is roughly the same for monopolar aDBS and bipolar aDBS.

## 4. Discussion

Pre-clinical and clinical achievements of closed-loop DBS in the treatment of PD attracted a lot of attention during the past decade (Little et al., 2013, 2016; Priori et al., 2013; Rosa et al., 2015, 2017; Johnson et al., 2016; Piña-Fuentes et al., 2017; Tinkhauser et al., 2017). One way for closed-loop control of pathologically synchronized neural activity within the parkinsonian BG is to monitor the collective activity of neurons in the target network (e.g., the STN) and adapt the stimulation amplitude (strength) to the level of neural synchrony (Tass, 2003; Popovych et al., 2017b; Popovych and Tass, 2019; Fleming et al., 2020a,b). Neural synchrony can be, for example, estimated by the large-amplitude oscillations of collective activity in a population of interacting oscillatory neurons. This idea was taken into account to develop a closed-loop aDBS for the treatment of patients with PD where stimulation delivery was modulated according to the level of STN beta band activity (Little et al., 2013, 2016), leading to a better improvement in motor symptoms while reducing the delivered stimulation current compared with cDBS (Little et al., 2013, 2016).

Here, we developed a comprehensive cortico-BG-thalamic network model to investigate the efficiency of closed-loop control of the aDBS amplitude in comparison with the open-loop cDBS. The parkinsonian network model was characterized by excessive beta oscillations within STN, GPe, and GPi and reduced thalamic reliability. Subthalamic aDBS effectively suppressed parkinsonian beta oscillations and restored normal range of firing activity (in STN, GPe, and GPi) and preserved thalamic reliability. STN aDBS led to better suppression of pathological beta oscillations while notably less stimulation current was delivered compared with cDBS. Particularly, aDBS with the same stimulation frequency as cDBS led to a better beta suppression efficiency (i.e., aDBS: 3.6% vs. cDBS: 1.7%), while the energy expenditure was 41% less than cDBS (see Figure 8). Interestingly, the value of thalamic reliability was similar for both stimulation protocols (i.e., aDBS: 53% vs. cDBS: 52%).

In computational models of PD, response failures of thalamo-cortical cell populations tend to coincide temporally, whereas under DBS, these failures, when they occurr, are temporally dispersed (Guo et al., 2008). To explore the effect of DBS frequency on the thalamic reliability, we calculated the error index introduced by Rubin and Terman (2004), defined as the total number of errors divided by the total number of input stimuli (Rubin and Terman, 2004; So et al., 2012; Alavi et al., 2022). In this context, the optimal performance is achieved when each sensorimotor input pulse results in a single action potential in a thalamic neuron. As shown previously, in a model developed by Rubin and Terman (2004), DBS above 20 Hz was effective at restoring the accuracy of thalamic transmission. Later, it was shown that stimulation below 40 Hz caused the rate of errors made by the thalamic cell to remain high, while stimulation above 100 Hz restored thalamic fidelity in a computational model of the BG (So et al., 2012). As shown in Supplementary Figure S2, our results show that aDBS above 100 Hz is effective at restoring the thalamic fidelity to its healthy level, with the best performance at 130 Hz.

In this study, the amplitude (strength) modulation in closed-loop control of the STN aDBS was performed by using the P controller scheme utilizing an LFP-derived measure of network beta band oscillatory activity (Fleming et al., 2020a,b), similar to that used during clinical closed-loop DBS protocols (Little et al., 2013, 2016). However, several studies employed alternative biomarkers for PD symptoms, such as entropy (Dorval et al., 2010; Dorval and Grill, 2014; Anderson et al., 2015; Syrkin-Nikolau et al., 2017), phase-amplitude coupling (De Hemptinne et al., 2013, 2015), coherence (Al-Fatly, 2019), and gamma band (30–80 Hz) activity-based measures (Swann et al., 2016, 2018). While amplitude modulation by the P controller utilizing LFP beta activity may not capture the neural mechanisms behind some of the parkinsonian symptoms and their specifically developed closed-loop DBS protocols, it may still be applicable to alternative stimulation methods, such as phase-based (Tass, 2003; Holt et al., 2016, 2019) linear delayed feedback (Popovych and Tass, 2019) and optogenetic (Detorakis et al., 2015) stimulation paradigms.

Taken together, closed-loop aDBS protocols with different stimulation frequencies led to better suppression of parkinsonian beta oscillations than open-loop cDBS while reducing the amount of delivered current and, thereby, may reduce potential stimulation-induced side effects (Baizabal-Carvallo and Jankovic, 2016; Pyragas et al., 2020). This suggests that closed-loop aDBS with amplitude modulation can efficiently maintain the beta band activity in the STN LFP below the target pathological level. As previously shown in several studies (Su et al., 2019; Fleming et al., 2020a,b), the suppression efficiency of closed-loop aDBS may depend on the stimulation frequency, controller type, and parameters. For instance, stimulation frequency modulation in closed-loop aDBS (instead of stimulation amplitude modulation) can effectively suppress abnormal beta oscillations, but it may also significantly increase the amount of administered stimulation current (Fleming et al., 2020a).

Moreover, another limitation of our model is that we tuned synaptic couplings and applied currents in the model to mimic parkinsonian beta band oscillatory activity within the cortico-BG-thalamic network, where cortical input was simplified as an external current. However, cortical input shapes rhythmic activity in the GPe-STN network in the PD state. Experimental findings suggest that the beta band oscillatory activity of the cortex and STN are significantly coherent and the beta band synchrony is notably increased between the GPe and STN as well as between the STN and the cortex following DA depletion (Sharott et al., 2005; Mallet et al., 2008). Computationally, excessive beta band oscillatory activity within the GPe-STN loop can be phase-locked to cortical beta inputs in PD models (Koelman and Lowery, 2019). Hence, our model may not be able to capture the complex network interactions leading to pathological beta oscillations in PD but still can reproduce suppression efficient characteristics of closed-loop aDBS compared with the open-loop cDBS.

Several experimental findings suggested that DA deficiency in PD can lead to exaggerated beta band (15–30 Hz) activity within the BG (Brown et al., 2001; Sharott et al., 2005; Mallet et al., 2008); however, the exact mechanisms underlying pathological beta oscillations remain poorly understood. Experimental and mathematical models have shown that beta oscillations can emerge from inhibitory interactions among striatal MSNs (McCarthy et al., 2011), increased levels of the striatal cholinergic drive (Kondabolu et al., 2016), or GPe-STN interactions (Brown et al., 2001; Holgado et al., 2010; Tachibana et al., 2011). Yet, abnormal beta oscillations may not appear until the advanced stages of PD and are supposedly correlated with the extent of progressive degeneration of nigral DAergic neurons (Asadi et al., 2022). The degree of neural beta oscillatory activity is related to the magnitude of the response of the BG to DAergic neurons rather than directly to the severity of the patients' symptoms (Weinberger et al., 2006). Variability in the symptoms of patients with PD suggests that neural beta oscillatory activity, alone, may not reflect the clinical state of the patient, and other complex mechanisms must be involved in the disease pathophysiology (Weinberger et al., 2006). For instance, it has been shown that administration of some drugs increases STN beta oscillations while decreasing tremor and rigidity (Priori et al., 2004) and that clinical improvement after DBS is not associated with an expected decrease in beta LFP activity in the STN (Foffani et al., 2006). While our model did not take into account patient-specific variability of abnormal beta oscillations, the development of customized patient-specific models of DBS in future studies may promote clinical improvements (Hollunder et al., 2022).

Intriguingly, a number of experiments failed to establish a significant correlation among PD motor symptoms, such as bradykinesia, akinesia and rigidity, and excessive beta oscillations during parkinsonism (Weinberger et al., 2006; Stein and Bar-Gad, 2013). In fact, abnormal synchrony in patients with PD has been observed in different frequency bands that can be related to different disease symptoms (Kühn et al., 2006; Weinberger et al., 2006; Steigerwald et al., 2008; Contarino et al., 2012). For instance, the presence of tremor in patients with PD has been linked to beta band (3–8 Hz) neural oscillations in the dorsal STN (Contarino et al., 2012). While, in some studies, synchronized beta band (15–30 Hz) oscillations in the STN were specifically attributed to the presence of tremor (Levy et al., 2000), others did not find any difference between PD patients with or without resting tremor in the frequency distribution of oscillatory neural activity when considering the entire frequency range of 1–100 Hz (Steigerwald et al., 2008). In the context of the choice of frequency band used as a biomarker for closed-loop aDBS, beta frequency oscillations in the LFP may capture variation in bradykinesia and rigidity across patients (Little and Brown, 2012), but this should be confirmed in each patient since it may impact the set of symptoms that can be suppressed by the presented aDBS approach (Little and Brown, 2012; Johnson et al., 2016). More importantly, biomarkers that reliably reflect other impairments, such as tremor, also need to be tested. Of note, beta band power may not be the best biomarker for closed-loop aDBS. For instance, a recent longitudinal study showed that although DBS significantly suppressed beta band activity, the suppression effect appeared to attenuate gradually during a long-term 6-month follow-up period after surgery (Chen et al., 2020). While long-term attenuation of DBS effects may be due to the progression of the disease or the stimulation protocol itself (i.e., cDBS vs. aDBS), the sensitivity and reliability of other frequency bands as potential biomarkers that are selective to different PD symptoms need to be investigated.

The presence of beta oscillations (15–30 Hz) within the BG may not be always pathological, and transient beta oscillations can be related to the normal activity of the motor system, such as the intention and initiation of movement (Little and Brown, 2014; Khanna and Carmena, 2017). However, beta oscillations are significantly enhanced in PD, and there is strong correlative evidence linking beta activity at rest to the changes in beta power in response to treatment in patients with bradykinesia and rigidity (Sharott et al., 2005; Mallet et al., 2008; Little and Brown, 2014). In our model, the stimulation has only been delivered during periods of elevated beta activity through the closed-loop aDBS protocol. Our model, therefore, ignores the selectivity of the abnormal beta activity and always suppresses the beta activity regardless of its causal or quantitative origin. It remains to be studied in future how normal and pathological beta oscillations can be distinguished and how stimulation delivery protocol can be improved, accordingly.

Our aim was to present a simple, yet comprehensive model of the BG. Therefore, we ignored the role of fast-spiking interneurons (FSIs) in the BG circuitry since they supposedly constitute < 5% of total striatal neurons (Koós and Tepper, 1999). However, as shown previously, the presence of FSIs may impact the emergence of strong synchronization and propagation of beta oscillations, which are a hallmark of parkinsonian circuit dysfunction (Corbit et al., 2016). Particularly, when GPe spikes are synchronous, the GPe-FSI pathway results in synchronous FSI activity pauses, allowing for a transient window of disinhibition for MSNs (Corbit et al., 2016). Accordingly, the inclusion of FSI into the BG circuitry in our model may affect the presented results by indirectly modulating the level of abnormal beta activity used as the biomarker of the disease.

In our study, the model parameters were extracted from the rodent models of PD. This might affect the impact of the aDBS protocol used in this study and need to be adopted for success in human clinical trials. Animal models may suffer from several limitations. For instance, in rodents, interventions may precede induction of the model and the outcomes may be less commonly assessed at multiple time points (Zeiss et al., 2017). Therefore, potential therapies for PD that are successful in animal studies may fail in human trials. The translational gap for potential therapeutic interventions in PD in part results from study designs that fail to model the progressive nature and relatively late intervention characteristic of PD (Zeiss et al., 2017). Yet, animal models enable the possibility to study the pathological mechanisms and the therapeutic principles of treating disease symptoms in humans. Once the causative mechanisms are clarified, animal models can be helpful in the development of therapeutic approaches and pave way for the transition from animal models to translational application in patients with PD.

Finally, abnormal synchronization is a hallmark of PD (Brown et al., 2001; Hammond et al., 2007). Such abnormal synchronization can be controlled by the administration of high-frequency desynchronizing brain stimulation to the diseased network (Popovych and Tass, 2014). However, the emergence of abnormal neural synchronization during parkinsonism cannot be solely ascribed to the pathological changes of neural dynamics following DA loss. Other complex mechanisms may be involved (Madadi Asl et al., 2018b, 2022b; Ziaeemehr et al., 2020). For instance, dysfunction of DA-mediated synaptic plasticity during parkinsonism shapes abnormal synaptic connectivity within the BG (Fan et al., 2012; Madadi Asl et al., 2019, 2022b). This further supports the emergence of pathological neural activity and synaptic connectivity patterns (Madadi Asl and Ramezani Akbarabadi, 2022) within the parkinsonian BG (Madadi Asl et al., 2022b). Thus, an effective brain stimulation technique should in fact decouple neurons (Madadi Asl et al., 2023), that is, desynchronize overly synchronized neural activity and reduce pathological synaptic connectivity to ensure long-lasting therapeutic effects that persist after stimulation offset (Madadi Asl et al., 2023).

In this study, the synaptic connections among neurons in the network model were assumed to be static, that is, the synaptic strengths were fixed in time. However, beneficiary long-lasting stimulation effects can be, in principle, achieved in neural network models of PD with plastic synapses modified by spike-timing-dependent plasticity (STDP) (Gerstner et al., 1996; Markram et al., 1997; Bi and Poo, 1998), as shown by computational studies (Tass and Majtanik, 2006; Hauptmann and Tass, 2009; Popovych and Tass, 2012; Lourens et al., 2015; Kromer and Tass, 2020). STDP can mold multistable neural and synaptic network dynamics (Madadi Asl et al., 2017, 2018a,c; Ratas et al., 2021) that can be computationally attributed to physiological and pathological basins of attraction (Madadi Asl et al., 2022b). In this way, appropriately tuned, STDP-targeting stimulation protocols can shift patterns of neural activity and synaptic connectivity in plastic networks from pathological states (characterized by strong synchrony and strong connectivity) to more physiologically favored states (characterized by weak synchrony and weak connectivity) (Madadi Asl et al., 2022b, 2023).

## Data availability statement

The original contributions presented in the study are included in the article/Supplementary material, further inquiries can be directed to the corresponding authors.

## Author contributions

AV and SS conceived and designed the study. FB-J performed the material preparation and numerical simulations. FB-J, SS, MM, and AV analyzed the results. MM wrote the first draft of the manuscript. All authors contributed to manuscript revision, read, and approved the submitted version.
