# Supplementary material for: Efficient suppression of parkinsonian beta oscillations in a closed-loop model of deep brain stimulation with amplitude modulation
Source: Front Hum Neurosci. 2023 Jan 26;16:1013155. doi: 10.3389/fnhum.2022.1013155 (PMC9908610; doi:10.3389/fnhum.2022.1013155)
Supplement: Supplementary file 1 [file Data_Sheet_1.PDF]

Supplementary Material

1 SUPPLEMENTARY FIGURES

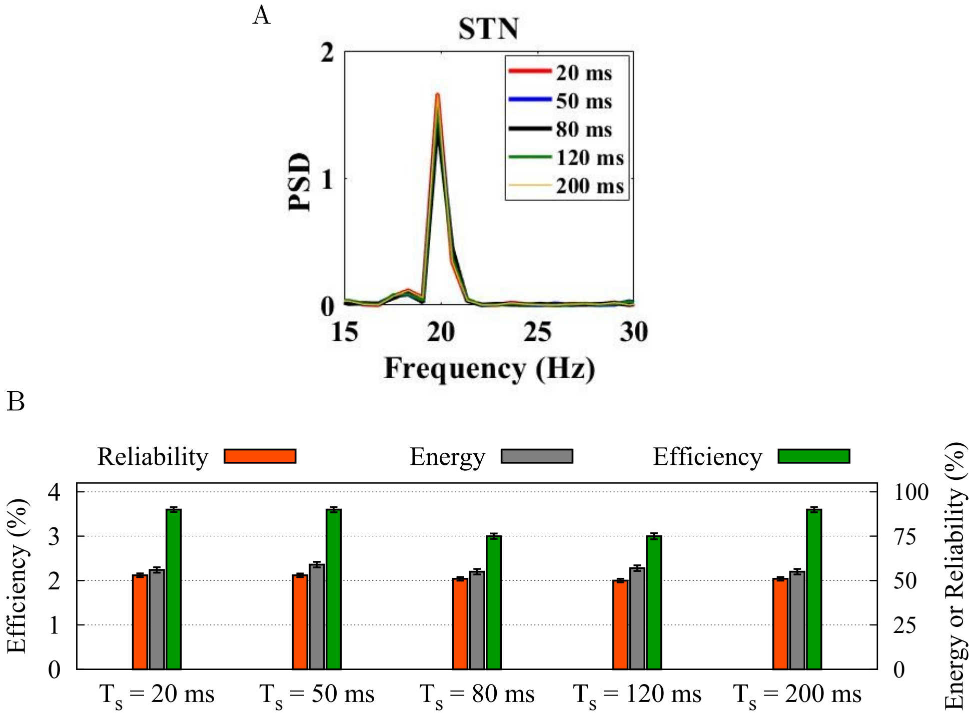

Figure S1. The effect of sampling time window on beta power measurement and stimulation performance. (**A**) Power spectrum density of the STN LFP activity assessed for different time windows, i.e., $T_s = 20, 50, 80, 120, 200$ ms. (**B**) The thalamic reliability (orange), the energy expenditure index (grey) and the beta suppression efficiency for the STN (green) for each sampling time window during 130 Hz aDBS. Standard deviation bars are shown for 10 simulations under each condition.

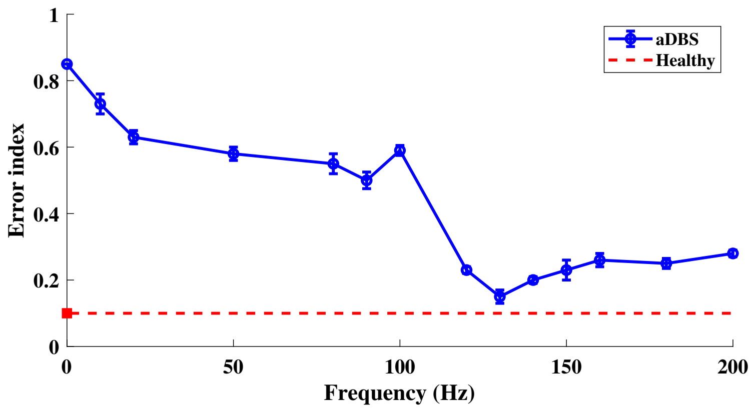

Figure S2. The effect of stimulation frequency on error index of the thalamic cells. aDBS above 100 Hz was effective at restoring the thalamic fidelity to its healthy level (denoted by dashed red line). Standard deviation bars are shown for 10 simulations under each condition.
